# Immunotherapy Decision-Making Experiences of Patients With Cancer and Their Family Caregivers: A Qualitative Study

**DOI:** 10.1097/jnr.0000000000000713

**Published:** 2025-11-12

**Authors:** Yun-Hsiang Lee, Chia-Li Siao, Zi-Xuan Gao, Cheng-Fang Yang

**Affiliations:** 1 School of Nursing, College of Medicine, National Taiwan University, Taipei, Taiwan; 2 Department of Nursing, National Taiwan University Cancer Center, Taipei, Taiwan; 3 Department of Oncology, National Taiwan University Hospital, Taipei, Taiwan; 4 Second Degree Bachelor of Science, College of Medicine, National Taiwan University, Taipei, Taiwan; 5 Department of Nursing, National Taiwan University Hospital, Yunlin Branch, Yunlin, Taiwan

**Keywords:** cancer, family caregiver, qualitative study, immunotherapy, decision-making

## Abstract

**Background:**

Patients with cancer and their families anticipate that innovative treatment options such as immunotherapy may improve survival and quality of life. Qualitative interviews have rarely been used to examine the immunotherapy-related decision-making experiences of cancer patients and their family caregivers.

**Purpose:**

This study was developed to explore the factors and reasons influencing the decisions of patients with cancer and their family caregivers to accept immunotherapy.

**Methods:**

Between October 2019 and January 2021, data were collected using in-depth interviews that were conducted face-to-face in a semistructured format and audio-recorded. Content analysis was performed, and the Consolidated Criteria for Reporting Qualitative Research checklist was applied.

**Results:**

Fifteen patients with cancer in Taiwan who had received immunotherapy and 17 family caregivers were enrolled as participants. Three main themes were common among the considerations of patients and caregivers with regard to their decision-making to undergo immunotherapy. These included (a) putting decisions in the hands of health professionals, (b) the value of life surpasses all other considerations, and (c) practical considerations such as treatment side effects, financial burden, and the effects of immunotherapy.

**Conclusions/Implications for Practice:**

The immunotherapy decision-making experiences of patients with cancer and their family caregivers in Taiwan were explored in this study. The generally low level of medical expertise identified among the participants emphasizes the importance of fostering a shared approach to immunotherapy decision-making. Also, oncology nurses should prioritize the inherent worth of patients and the emotions of their family caregivers to bolster their confidence and sense of self-worth. Notably, experiencing severe side effects with previous treatments was found to be a strong predictor of immunotherapy choice, highlighting the critical importance of regular assessment of patients’ immunotherapy experiences in clinical practice. Also, financial concerns were confirmed as an important factor affecting patient-centered care. Thus, when needed, additional financial support should be provided.

## Introduction

Patients with cancer and their families anticipate that innovative treatment options such as immunotherapy can effectively manage their disease progression (Emens et al., 2016), with major enhancements in overall survival achieved for patients with certain types of cancer (Farrow et al., 2020; Putzu et al., 2023; Reck et al., 2021). In a study by Reck et al. (2021) on patients with non–small cell lung cancer (NSCLC) and a PD-L1 (programmed cell death-ligand 1) tumor proportion score of ≥50%, the survival rate in the group treated with pembrolizumab (31.9%) was significantly higher than that in the group treated with chemotherapy (16.3%). Moreover, the subset of these patients (25.3%) who received pembrolizumab for approximately 2 years (approximately 35 cycles) attained a 5-year survival rate of 82.1% (Reck et al., 2021). Clinical trials are facilitating the rapid advance of immunotherapies (Esfahani et al., 2020; Kruger et al., 2019), instilling in patients with cancer the hope of survival (Nouri Rouzbahani et al., 2018). As a result, patients with cancer and their families are increasingly faced with making decisions related to immunotherapy.

In cancer treatment, decision-making refers to the process by which patients, often in collaboration with their health care providers and family members, evaluate available information, including the potential benefits and risks of each treatment option, and ultimately make informed decisions regarding the implementation of anticancer therapy for disease management (Josfeld et al., 2021). With the increased use of immunotherapeutic agents in cancer treatment, the Oncology Nursing Society has emphasized the key role played by oncology nurses in immunotherapy, establishing guidelines for nurses responsible for monitoring patients undergoing immunotherapy that address the proper handling of immunotherapy agents and ensure patient safety (Wiley et al., 2017). Also, the key role played by nurses in shared decision-making has been highlighted in terms of providing the critical support necessary to meet patient needs, guiding crucial decisions, and applying their medical knowledge and understanding of patient preferences as effective partners in the decision-making process (Karina et al., 2021).

Although increasing numbers of patients with cancer are undergoing immunotherapy (Das & Johnson, 2019; Lee et al., 2022), only some of these patients realize long-term survival benefits from their treatments (Reck et al., 2021). In addition, a consistently low overall response rate (15%–30%) has been reported for various types of solid tumors (Das & Johnson, 2019). In many countries, including Taiwan, immunotherapy is expensive. The National Health Insurance program of Taiwan covers immunotherapy only for those cancer types, including melanoma, NSCLC, urothelial carcinoma, and head and neck squamous cell carcinomas, that meet the PD-L1 expression criteria (Lee et al., 2022). Patients with metastatic NSCLC who undergo either immunotherapy or chemoimmunotherapy also face significant financial burdens (McLouth et al., 2021). Although research on immunotherapy has often focused on treatment responses and survival outcomes, the uncertainty of long-term survival combined with the high costs of treatment can cause psychological stress for patients. In Japan, 7.5% of grieving family members reported discontinuing or modifying cancer treatments due to financial hardships, highlighting the link between financial strain and treatment decisions (Sasaki et al., 2022).

Despite these challenges, some patients still choose immunotherapy. What drives these decisions? Limited research into how patients and their family caregivers make these choices has been conducted. Family caregivers influence cancer treatment decision-making through helping patients gather information, identifying important decisions, and discussing their values and illness perceptions (Dionne-Odom et al., 2019). Among Chinese cancer survivors, in particular, family members have been shown to significantly impact treatment decision-making (Wang et al., 2020). In previous studies, treatment decisions have been shown to depend strongly on physician recommendations, with patients giving these recommendations significant weight (Ihrig et al., 2020). Given the complexities involved in making immunotherapy-related decisions, this study was developed to examine the perspectives of both patients and their caregivers to better understand the factors influencing these decisions.

## Methods

### Research Design

A qualitative design was used to investigate patient and caregiver decision-making regarding immunotherapy. In-depth interviews were conducted to allow the participants to articulate their experiences fully. The objective of qualitative research is to ascertain the internal thoughts and experiences of individuals to elucidate a deeper understanding of human experiences, which is crucial for health care professionals to enhance the quality of their care, communications, and interactions (Holloway & Galvin, 2016; Speziale et al., 2011). An inductive model was employed to analyze collected data using content analysis (Graneheim & Lundman, 2004).

### Participants and Settings

Patients undergoing immunotherapy at oncology wards in a medical center in Taiwan were recruited as participants between October 2019 and January 2021. The caregivers of these patients were also invited to participate. However, eligible patients were allowed to enroll as participants alone Purposive sampling was used. Eligibility criteria for patient participants included: having a physician-confirmed cancer diagnosis, being ≥20 years old, having undergone immunotherapy, and having received ≥1 dose of immunotherapy drugs. Otherwise eligible patients diagnosed with a mental disorder, such as depression or anxiety, who were not able to communicate effectively or who refused to participate, were excluded. Family caregivers who were ≥20 years old, lived with the patient, and were the primary caregiver were invited to participate. However, those unable to communicate effectively were also excluded.

### Data Collection

Data were collected using a semistructured interview form developed based on prior empirical research (Hosseini et al., 2021; Joseph-Williams et al., 2014; Josfeld et al., 2021; Speziale et al., 2011) and discussions with experienced oncologists.

The participants were asked the following questions:

- Where did you hear about immunotherapy? (Patient/caregiver)
- Please tell me your opinion on immunotherapy for cancer. (Patient /caregiver)
- Which therapies did you undergo before immunotherapy, and how was your experience with these therapies? (Patient)
- What prompted your decision to (undergo/support) immunotherapy? (Patient/caregiver)
- How have you been feeling since the start of immunotherapy? (Patient)
- What expectations and hopes do you have regarding immunotherapy? (Patient/caregiver)
- Is there anything else you would like to share with me or add? (Patient/caregiver)

The interviews were conducted by the first author (with over 10 y of experience in teaching oncology) and the third author (a skilled researcher with 5 years of oncology nursing experience). The third author was not working in the oncology ward where the interviews took place, which minimized the potential for bias while ensuring familiarity with the context. Before their interviews, all of the participants signed an informed consent form. The first and third authors interviewed each patient and caregiver individually before conducting the formal interview. They then held a face-to-face meeting to discuss the challenges encountered during the interview process and the questions asked. Based on this discussion, the first and third authors revised the interview guidelines to ensure consistency across interviews. The interview strategy used open-ended questions and minimized the number of leading questions. For example, the question “What are the physical symptoms or side effects experienced since starting immunotherapy?” was revised to “How have you been feeling since the start of immunotherapy?” to encourage a more open response. The interviewer employed face-to-face, open-ended questioning techniques to elicit responses from the participants. Before the interviews, potential participants were informed about the study's purpose and procedures. They confirmed their willingness to participate and chose the interview time and location, helping ensure they would express their feelings comfortably and honestly. Participants consented to the interviews, which lasted between 40 and 90 minutes, being audio-recorded. Data collection ended when all relevant research topics had been sufficiently analyzed and no new information or concepts emerged.

### Data Analysis

The obtained interview recordings were transcribed by the two interviewers within 2 weeks of the actual interviews. Next, content analysis was performed (McLouth et al., 2021). Initially, the corresponding author and interviewers independently and repeatedly read the transcripts to gain a comprehensive understanding of the content and establish units of analysis. Following this, all of the statements regarding the thoughts, perceptions, and responses of both participants and caregivers regarding immunotherapy decision-making were extracted. These statements were assigned relevant meaning units independently by the corresponding author and the first author. Then, the first and corresponding authors collaboratively discussed the individual analyses of the patient interviews, resolved any discrepancies, and reviewed the interview text repeatedly until consistency in categorization and themes was achieved, ensuring the analysis accurately reflected the feelings and experiences of the participants. The same approach was subsequently applied to the analysis of the caregiver interviews. Next, the first and corresponding authors reviewed the analyses of categorization and themes from patients and caregivers side by side to identify any differences. The findings revealed that patients and caregivers had conveyed nearly identical experiences and perceptions regarding decision-making about immunotherapy.

### Rigor

Data trustworthiness was assessed using several strategies (Graneheim & Lundman, 2004; Lincoln & Guba, 1985). To establish credibility, the third author, a registered nurse with over 5 years of experience caring for patients with cancer in the same oncology ward where the interviews were conducted, underwent training in conducting qualitative interviews. The first author, an adjunct supervisor at the study hospital, regularly spends 1 month each year in the admissions ward supervising nursing students responsible for providing oncology patient care. To minimize data collection bias, a standardized interview guide and field notes were employed to account for potential influences, including the dual role of caregivers as researchers, in the analysis. Peer debriefing was conducted to validate the emergent themes and patterns against the data collected. Rich descriptions and data analyses were conducted individually by the first author and the corresponding author, followed by discussions to ensure the confirmability of the findings. Dependability was assessed using field notes and meticulous data analysis procedures. Furthermore, thick descriptions of the research context, field notes, and methodology (e.g., using purposive sampling to clarify the transferability and generalizability of findings to analogous situations) were provided.

### Research Ethics

This study was approved by the research ethics committee of the participating hospital (IRB No. 201812191RINB; Clinical Trials Number: NCT05740800), and informed consent was obtained from all of the participants. Participation was voluntary, and participants were informed they could discontinue their interview at any point. Participant anonymization was employed to maintain and protect data confidentiality. Finally, this study was conducted in accordance with the Consolidated Criteria for Reporting Qualitative Research checklist (Tong et al., 2007).

## Results

Fifteen patients and 17 family caregiver participants were interviewed. The interviewed patient participants had a mean age of 58 years, 80% were male, 93.3% were married, 46.7% had completed high school only, 20% were employed at the time of the interview, and 40% had a diagnosis of hepatocellular carcinoma (Table 1).

**Table 1:** Characteristics of Patient Participants (*N*=15)

ID	Age	Gender	Diagnosis	Stage	Associated Decision-Making Theme(s)		
					1	2	3
1	24	Male	Hepatocellular carcinoma	IV	V		
2	65	Female	Lymphoma	IV	V	V	V
3	52	Male	Hepatocellular carcinoma	IV	V		
4	70	Male	Thyroid cancer	IV	V	V	
5	62	Male	Hepatocellular carcinoma	II	V		V
6	50	Female	Lung cancer	III	V		
7	45	Male	Melanoma	IV	V	V	V
8	42	Male	Nasopharyngeal carcinoma	IV		V	V
9	69	Male	Lung cancer	II		V	
10	65	Male	Hepatocellular carcinoma	IV	V		
11	70	Female	Hepatocellular carcinoma	IV		V	V
12	59	Male	Hepatocellular carcinoma	IV	V	V	
13	76	Male	Pancreatic cancer	IV	V	V	
14	55	Male	Colon cancer	IV	V	V	
15	66	Male	Hepatic angiosarcoma	IV		V	V
	Mean 58	Male 80%	Hepatocellular carcinoma 40%	IV80%			

*Note*. 1 = putting decisions in the hands of health professionals; 2 = the value of life surpasses all other considerations; 3 = practical considerations.

With regard to the family caregiver participants, a majority were female (94.1%), 76.5% were married, 41.2% had completed high school only, and another 41.2% had completed college, 64.7% were employed at the time of the interview, and 12 (70.6%) were married to their paired patient. Three-quarters (76.5%) of the family caregiver participants cared for their patient from 13 to 24 hours per day (Table 2).

**Table 2:** Characteristics of Family Caregivers Participants (*N*=17)

ID	Age	Gender	Relationship With Patient	Associated Decision-Making Theme(s)		
				1	2	3
1	50	Female	Child	V	V	V
2	48	Female	Sister	V		V
3	51	Female	Spouse		V	
4	56	Female	Spouse	V	V	
5	75	Female	Spouse	V	V	V
6	64	Female	Spouse	V	V	V
7	59	Female	Spouse		V	V
8	41	Female	Spouse	V		V
9	63	Female	Spouse		V	V
10	27	Female	Granddaughter			V
11	35	Female	Spouse			V
12	64	Female	Spouse		V	V
13	50	Female	Child			V
14	51	Female	Spouse	V		V
15	71	Female	Spouse			V
16	49	Male	Child	V		V
17	65	Female	Spouse	V	V	V
	Mean 54.1	Female 94.1%	Spouse 70.6%			

*Note*. 1 = putting decisions in the hands of health professionals; 2 = the value of life surpasses all other considerations; 3 = practical considerations.

The decision-making process used by patient participants with regard to immunotherapy was found to be highly similar to their paired caregivers. The pertinent considerations were (a) putting decisions in the hands of health professionals, (b) the value of life surpasses all other considerations, (c) practical considerations such as treatment side effects, financial burden, and the effects of immunotherapy (Table 3).

**Table 3:** Immunotherapy Decision-Making Experiences of Patient and Family Caregiver Participants

Theme	Category	Example of Quotes
1. Putting decisions in the hands of health professionals	(1) Necessity of cooperating with physicians due to limited medical expertise	To be honest, I’m not a medical professional. What else can I do? There’s nothing I can do on my own. The doctors said the immunotherapy would be good for me, so I can only trust their advice and follow it. (Case 12)
	(2) Trusting physician decisions	We completely follow the doctor’s advice. Whatever the doctor suggests, we do exactly as advise. Yes, we rely completely on the doctor to tell us what to do. (Case 2)
2. The value of life surpasses all other considerations	(1) Take any opportunity for survival	…I have to take medicine in order to recover from the illness quickly… having rashes is a minor issue… getting better from the illness is the most important thing… (Case 4)
	(2) Life is worth more than money	He worked so hard on his whole life… it’s worth using (immunotherapy) on him…money is not problem…this is his last chance (Caregiver 4)
3. Practical considerations	(1) Unable to endure the side effects of existing treatment	Since chemotherapy isn’t effective and causes my dad to feel exhausted and lose his hair, immunotherapy seems like a better option. We recently came across an opportunity to join a clinical trial, and my dad has finally agreed to participate. (Caregiver 16)
	(2) Consider the financial burden of immunotherapy	This is how I see it—I didn’t tell him (the patient) (laughs). In the future, if I run out of money, I won’t be able to afford to live, and I’ll have to stop the immunotherapy (Caregiver 5)
	(3) Looking forward to the effects of immunotherapy	Is immunotherapy good or not? We just have to try it. Financially, it’s not too much of a burden, but there’s still the worry about its effectiveness (Caregiver 9)

### Putting Decisions in the Hands of Health Professionals

Both patient and caregiver participants expressed significant concerns about immunotherapy, primarily due to limited medical knowledge and reliance on the expertise of attending physicians.

#### Necessity of Cooperating With Physicians Due to Limited Medical Expertise

Many of the patient participants expressed the belief that their doctor knows best and that their own knowledge is insufficient to make properly informed decisions. Seven patients (47%) and six (35%) caregiver participants reported a lack of medical knowledge, making them unable to follow the advice of health care professionals. One of the patient participants, a 42-year-old with nasopharyngeal carcinoma who had received chemotherapy for 2 years with no improvement in symptoms, reported being advised 6 months ago by their physician to try immunotherapy. The patient said:

*We don’t know much about healthcare ourselves so, naturally, we were quite alarmed the first time we encountered it* (immunotherapy). *We’re already unwell, and we came here to entrust our care to a professional because, otherwise, there’s nothing we can do!* (Case 8)

Another patient participant, a 59-year-old man who had been diagnosed with hepatocellular carcinoma 2 years earlier, had been advised to undergo immunotherapy due to his high alpha-beta protein levels. He said:

*To be honest, I don’t really understand healthcare, so I just have to trust the professionals - it’s as simple as that. After all, the doctors know; but how do I know what treatments will come next? Many doctors say that immunotherapy and targeted therapy are very effective against cancer cells.* (Case 12)

Similarly, due to their unfamiliarity with the medical profession, the only way forward for many of the participants was perceived to be trusting their health care providers. One of the caregiver participants stated:

*…Because we don’t understand medicine, there’s no need to scare ourselves; just cooperate with the treatment, and remember that not everyone experiences the same side effects.* (Caregiver 4)

#### Trusting Physician Decisions

During the interviews, six patient and eight caregiver participants expressed confidence in the professionalism of their doctors and accepted their recommendations regarding immunotherapy. One of the patient participants with hepatocellular carcinoma said:

*My attending physician is highly qualified and very reliable, so I trust my treatment to Dr Xu*. (Case 10)

Another shared that she had always followed her doctor’s advice, believing she should simply trust him. She said:

*I listen to the doctors, follow their advice, cooperate with them, and trust their expertise while also doing what I believe is right for myself*. (Case 11)

A wife caring for her husband, a patient participant with pancreatic cancer, accepted the physician’s suggestion of chemotherapy combined with immunotherapy. She said:

[The doctor said] *there is a new type of immunotherapy… It may be better for his body when combined with chemotherapy… We just follow the doctor’s advice… we really don’t know which medications are best; only the doctor does… If he says it can be administered, then it should be okay*. (Caregiver 6)

Another caregiver participant stated they simply follow the doctor’s advice and don’t trouble themselves with additional concerns.

*We just listen to what the doctor says and do whatever he recommends, so it doesn’t seem to bother us particularly* *…* (Caregiver 5)

### The Value of Life Surpasses All Other Considerations

Facing death is a frightening experience. Patients with cancer find it difficult to accept the thought of losing their health and approaching death, with some willing to take all opportunities to survive and some perceiving life as worth more than money.

#### Take Any Opportunity for Survival

A cancer diagnosis can be devastating, and deciding on the appropriate treatment can be a complex and daunting task. The majority of the patient participants expressed being willing to try every treatment option available to them. A 45-year-old patient participant with melanoma expressed that treatment effectiveness was essential for him because treatment gave him another chance to live longer.

*I am concerned about the curative effect… Regardless of whether a new drug is available or not, as long as the drug can kill cancer cells, I am willing to try any treatment*. (Case 7)

Another patient participant, after undergoing surgery for thyroid cancer, had received chemotherapy for over 10 years with poor control over her condition and had received immunotherapy for 2 years before the study. She said:

*I took the targeted medication, but it didn’t work, so I thought there must be a way to cure it—I just didn’t know which one. Then the doctor told us about immunotherapy, so I discussed it with my husband, and we decided to give it a try*. (Case 11)

Another patient participant who had undergone unsuccessful chemotherapy and radiotherapy chose to undergo immunotherapy as a new treatment option. He said:

*I’ve had chemotherapy and radiotherapy, but they didn’t work, and I relapsed. My doctor recommended immunotherapy, the latest treatment, to see if it might be effective for me. So, I have to seize the opportunity, and give it a try*. (Case 2)

Also, one of the patient participants relayed that he had chosen immunotherapy in hopes of starting treatment earlier. He said:

Anyway, it’s somewhat of a semi-forced decision. I was originally scheduled to start chemotherapy in mid-November, but then I was told that if I joined this immunotherapy program, I could begin earlier–at the end of October. (Case 8)

#### Life Is Worth More Than Money

Many people hold the belief that life is priceless and that no one has the right to make decisions regarding the life of another individual. In this study, the caregiver participants considered the value of life and respect for the patient’s wishes, which are factors that go beyond money. One caregiver participant, a wife caring for her 68-year-old husband with lung cancer, said:

*The doctor said the standard treatment abroad involves combining chemotherapy and immunotherapy… He* (the patient) *also insisted on undergoing immunotherapy because he wants to live, and we* (his wife) *must cooperate… As long as the treatment is effective, it’s worth spending the money…* (Caregiver 12)

Similarly, a caregiver participant who was caring for her husband with hepatic hemangiosarcoma chose life after weighing the importance of money and life. She said:

*We considered the financial issue once, but then realized the value of life to be greater than money…so we decided he should undergo immunotherapy*. (Caregiver 17)

### Practical Considerations

Treatment may represent a final hope for patients with cancer. Therefore, when they have the opportunity to choose a treatment method, most patients and caregivers consider key factors such as side effects, financial burden, and expected effectiveness.

#### Unable to Endure the Side Effects of Existing Treatment

Deciding on the type of treatment to undergo involves considering a multitude of factors, and treatment side effects are among the most frequently considered. Oncological treatments, such as chemotherapy, radiation therapy, and targeted therapy, can induce numerous distressing physical and mental side effects. Two patients reported being unable to tolerate the side effects of chemotherapy. A patient with nasopharyngeal carcinoma who had undergone chemotherapy for 6 years said

*I took the initiative to talk to my doctor about immunotherapy…because I couldn’t stand the side effects of chemotherapy. Just the thought of going in for about going in for chemotherapy, I would have a panic attack!* (Case 8)

A patient with hepatocellular carcinoma had undergone chemotherapy for 2 years, with poor results, and experienced numerous side effects. Following the physician’s advice, the patient chose immunotherapy in the hope of reducing treatment side effects. He said

*Previous chemotherapy was ineffective, and the side effects made me feel uncomfortable. Therefore, I decided to follow the doctor’s recommendation and undergo immunotherapy*. (Case 15)

When the caregivers of patients with cancer witness their loved ones enduring the side effects of cancer treatments, they often experience stress and grief. In this study, seven caregivers chose immunotherapy for this reason. A woman who had been caring for her father, a patient participant with a renal cell carcinoma diagnosis of many years, said she could not bear to witness her father experiencing the side effects of his initial cancer treatment. After discussion with her family, she decided to let her father switch to immunotherapy, in the hope of reducing treatment side effects. She shared:

*My dad has kidney cancer… The side effects of his targeted drugs were very serious… Nausea, vomiting, diarrhea, and skin rash… The doctor has offered a program for us to try immunotherapy… I’m hoping the side effects won’t be as severe and that his body won’t feel so uncomfortable.* (Caregiver 13)

A caregiver participant who had been taking care of his grandfather (a patient with lung cancer) said:

*We consider his* (the patient’s) *body can afford* [to experience side effects] *… After discussing with family members, we decided to do immunotherapy because immunotherapy sounds less terrible than chemotherapy* [and has fewer side effects]. (Caregiver 10)

#### Consider the Financial Burden of Immunotherapy

Taiwan’s national health care insurance system ranks as one of the world’s best and has alleviated the financial burden of numerous patients. Thus, the costs of specific treatments affect the treatment decisions of patients and their families indirectly. In line with this situation, most of the patient participants mentioned financial burden as an issue, while caregiver participants highlighted issues adjacent to financial burden in their responses. A patient participant with colon cancer had undergone various treatments, including chemotherapy, targeted therapy, and radiation therapy, for more than 10 years with suboptimal efficacy. His physician suggested immunotherapy. He shared:

*What I’m more worried about is… money…* [The doctor] *said this kind of immunotherapy… will not be so expensive… Then I said, “Okay, I will give it a try.”* (Case 14)

Another patient participant waited for immunotherapy to become more affordable before considering it. He said:

*I learned about immunotherapy three years ago; but at that time, the cost was over $8 million, which was too expensive. Later, the price dropped to $800,000, so I told my doctor I wanted to try it.* (Case 8)

Similarly, a caregiver participant stated their family had considered immunotherapy after the National Health Insurance Administration approved related subsidies. They shared:

*We started with chemotherapy because it did not require any additional expense. Later on, the news reported the Bureau of National Health Insurance had approved subsidies for immunotherapy, so we decided to give it a try.* (Caregiver 11)

Another caregiver participant said:

*Because there was an opportunity to join this trial, we decided to try immunotherapy. If we had to pay for it out of pocket, I would have given up and likely opted for targeted drug therapy instead.* (Caregiver 7)

#### Looking Forward to the Effects of Immunotherapy

The treatment choices of patients reflect their values, disease understanding, and expectations regarding the outcomes of selected treatments. Immunotherapy is a relatively new treatment in Taiwan, and its effectiveness remains a key consideration for patients, particularly when other treatments have failed. One patient participant said:

*I care about efficacy—I’m not concerned about side effects. What matters most to me is how effective it is!* (Case 7)

Another patient participant who had experienced good results from the first immunotherapy session was willing to undergo the treatment again despite the continued side effects.

*I have to say, I’m satisfied that immunotherapy is keeping my tumors under control. Even if the side effects of the second injection are similar to this one, I will still continue with immunotherapy!* (Case 5)

Under this theme, some of the caregiver participants expressed concerns regarding the effects of immunotherapy, highlighting the patients’ suffering from cancer treatments. A caregiver participant taking care of a patient with pneumonia said:

*As long as the treatment is effective, it is worth the money; but I am afraid the money will be spent without any effect.* (Caregiver 12)

Similarly, another caregiver participant expressed:

*We are not concerned about the money…In fact, the main issue is whether the immunotherapy is effective or not.* (Caregiver 15)

## Discussion

The immunotherapy decision-making experiences of a sample of patients with cancer and their family caregivers were explored in this study. All of the patient participants had been recommended by their physicians to receive immunotherapy, which prompted them and their caregivers to consider and make related treatment decisions. After conducting a content analysis of interview transcripts, three primary themes were identified, including (a) putting decisions in the hands of health professionals, (b) the value of life surpasses all other considerations, and (c) practical considerations.

Immunotherapy, a relatively new cancer treatment, has gained prominence over the past decade. Compared with chemotherapy and radiotherapy, public awareness of immunotherapy remains limited, which often encourages patients to rely heavily on physician guidance for their treatment decisions (Noteboom et al., 2021). In this study, some of the patient and caregiver participants, lacking alternative options and sufficient medical knowledge, chose to trust and follow their physician’s advice, which is consistent with earlier research showing that patients often simply trust their physicians to make informed decisions (Joseph-Williams et al., 2014; Shokar et al., 2018), as reflected in the category “Trusting doctors’ decisions.”

These findings emphasize the need to enhance education for both patients and family caregivers. By offering easy-to-understand and actionable information on treatment efficacy, potential side effects, and clinical trial outcomes, patients may be better empowered to make more-informed decisions and reduce their willingness to simply accept physician-given recommendations (Josfeld et al., 2021; Lee et al., 2023). Furthermore, oncology nurses play a pivotal role in bridging this knowledge gap. As trusted health care professionals, they may act as advocates and communicators, offering personalized support and facilitating shared decision-making among patients, patient family members, and physicians (Tariman & Szubski, 2015). The findings of this study also underscore the important role of familial support in decision-making. Patients often seek insights from trusted others to understand the effectiveness and risk–benefit balance of treatments (Reyna et al., 2015). Similarly, family caregivers are critical partners in this process, helping contextualize medical information and provide emotional support. To strengthen decision-making, physicians should allow patients time to discuss treatment options with their families and draw from shared experiences. This inclusive approach can improve comprehension, reduce uncertainty, and ensure decisions align with patient values and preferences (Lee et al., 2023).

In this study, the strong desire of patient participants to survive was an important positive factor affecting their decision to pursue immunotherapy treatments. As humans naturally fear death and strive for survival (Ahrensdorf, 2000), this desire to survive is closely linked to the psychological need to maintain hope (Boulanger et al., 2025). Family caregivers also play a crucial role in treatment decisions (Laidsaar-Powell et al., 2017; Oncology Nursing Society [ONS], 2023; Yang et al., 2023), often prioritizing the value of life and respect for the patient’s wishes over financial concerns. Notably, slightly over half (53%) of the caregiver participants in this study reported their decisions as being guided primarily by moral and ethical considerations rather than monetary considerations. This suggests caregivers view their responsibility as not only practical but also deeply emotional and ethical. Caregivers frequently face difficult choices in balancing the potential benefits of treatment with the impact on the patient’s quality of life. These decisions are particularly influenced by cultural values that emphasize respect for patient wishes even in the face of challenging financial and emotional burdens. Thus, oncology nurses should prioritize the inherent value of their patients’ lives and consider the emotional and ethical concerns of caregivers. These nurses should aim to meet both the physical and psychological needs of patients and caregivers, uphold their rights and dignity, and be sensitive to cultural considerations (Bafandeh Zendeh et al., 2022; ONS, 2023; Tariman & Szubski, 2015; Yang et al., 2023; Yen et al., 2023). Also, they should empower patients to engage in self-advocacy, particularly during decision-making discussions about immunotherapy that involve family caregivers (Alsbrook et al., 2022).

Furthermore, the patient and family caregiver participants in this study all chose immunotherapy based on practical considerations such as the inability to bear the side effects of current treatments, financial burden, and hope for the potential benefits of immunotherapy. Consistent with prior research, many of the patient participants in this study chose immunotherapy after experiencing the debilitating side effects of chemotherapy treatments (Magee et al., 2020). The results of a recent systematic review indicate that only 16.5% of immunotherapy patients experience adverse events (AEs) at or above grade 3 and that 41.1% of chemotherapy patients experience AEs at or above grade 3 (Magee et al., 2020). Despite uncertainties regarding the effectiveness of immunotherapy, these side effects prompted the patients in this study to seek alternative treatments. Similarly, the financial burden of immunotherapy played a significant role in decision-making in this study, with caregivers weighing the cost of treatment against its potential benefits. Financial strain exacerbates the emotional challenges of decision-making, underscoring that health care providers must consider both the economic and psychological burdens of treatment on families.

In this study, none of the patient participants met the PD-L1 expression criteria necessary to qualify for immunotherapy coverage under Taiwan’s National Health Insurance program. Also, although approximately 73.3% of patients with cancer in Taiwan have personal insurance, these policies typically do not fully cover treatment costs. When faced with substantial expenses, patients and their families must consider the financial impact of treatment, with improved survival outcomes acting as a key factor influencing treatment decisions. Consistent with previous studies (Corn et al., 2022; Sarradon-Eck et al., 2023), the findings of this study indicate that caregivers prioritize respecting the wishes of their patients over financial concerns out of a sense of moral responsibility and emotional connection. This reflects the complex nature of decision-making, requiring caregivers to balance ethical, emotional, and practical considerations. Cultural values were also found to be pivotal. As reflected in our findings, respect for a patient’s wishes and familial responsibility are central considerations in Taiwan, encouraging caregivers to prioritize these factors over others (Gazaway et al., 2024). Health care professionals, particularly oncology nurses, should recognize these multifaceted influences on decision-making and offer support that addresses both the clinical and emotional needs of patients and their families (Bafandeh Zendeh et al., 2022). Therefore, oncology nurses should collaborate with medical teams to ensure that patients make well-informed treatment decisions by considering not only the clinical aspects but also the financial implications of treatment (Karina et al., 2021). Thus, the provision by social workers of targeted financial guidance and/or support is essential (Smith et al., 2022).

### Limitations

This study is affected by several limitations. First, the findings may not be generalizable to similar populations (i.e., patients with cancer and their family caregivers) in other countries, as the data analyzed in the present study were collected from one hospital in northern Taiwan only. Second, as most of the patient participants in this study were male (80%), the results may not be generalizable to female patients with cancer. Despite these limitations, a notable strength of this study is that the viewpoints of both patients with cancer and their family caregivers with respect to their choice to undergo immunotherapy were examined simultaneously. These findings contribute to the literature on treatment decision-making processes in Taiwan. Furthermore, the authors adhered to qualitative research standards and followed the Consolidated Criteria for Reporting Qualitative Research checklist to ensure accurate reporting.

### Conclusions

The three key themes in the immunotherapy decision-making process used by patients with cancer and their family caregivers identified in this study include reliance on health care professionals, prioritizing life over other considerations, and practical considerations such as treatment side effects, financial constraints, and the effects of immunotherapy. These findings suggest that empowering patients and caregivers with appropriate practical information and support can enhance shared decision-making and reduce reliance on physicians. Oncology nurses play a vital role in bridging knowledge gaps, providing culturally sensitive support, and addressing ethical and emotional concerns. Moreover, addressing the psychological impact of the severe side effects of prior treatments and providing financial resources when needed are essential components of patient-centered care. Adopting and implementing a holistic approach that incorporates these principles and measures will ensure treatment decisions align with patient values and improve overall care outcomes.

### Nursing Implications

The decision-making experiences of patients with cancer and their family caregivers with regard to choosing to undergo immunotherapy were examined and described in this article. Effective communication among oncology nurses, patients with cancer, and their family caregivers should be established to enable nurses to evaluate the decision-making experiences of the patients and caregivers and identify gaps in their understanding of immunotherapy. Also, oncology nurses should create a list of potential treatment side effects to ensure both patients and their family caregivers understand the risks and help them understand and potentially ameliorate subsequent side effects effectively. Moreover, oncology nurses may work to help alleviate the high financial burden of immunotherapy on these patients by coordinating with physicians and social workers to explore financial support options, ensuring eligible patients remain able to access effective immunotherapy treatments.
